# Anticoagulation increases alveolar hemorrhage in mice infected with influenza A

**DOI:** 10.14814/phy2.13071

**Published:** 2016-12-21

**Authors:** Kohei Tatsumi, Silvio Antoniak, Saravanan Subramaniam, Bertrand Gondouin, Scott D. Neidich, Melinda A. Beck, Jacqueline Mickelson, Dougald M. Monroe, Julie A. Bastarache, Nigel Mackman

**Affiliations:** ^1^ Department of MedicineDivision of Hematology and OncologyUNC McAllister Heart InstituteUniversity of North Carolina at Chapel HillChapel HillNorth Carolina; ^2^ Department of Pathology and Laboratory MedicineUniversity of North Carolina at Chapel HillChapel HillNorth Carolina; ^3^ Department of NutritionUNC Gillings School of Global Public HealthUniversity of North Carolina at Chapel HillChapel HillNorth Carolina; ^4^ Department of MedicineDivision of AllergyPulmonary and Critical Care MedicineVanderbilt University School of MedicineNashvilleTennessee; ^5^ Present address: Department of Physiology and Regenerative MedicineKindai University Faculty of Medicine377‐2 Ohno‐higashiOsaka‐sayama, Osaka589‐8511Japan

**Keywords:** Alveolar hemorrhage, anticoagulation, influenza A virus infection, mouse model

## Abstract

Influenza A virus infection is a common respiratory tract infection. Alveolar hemorrhage has been reported in patients with influenza pneumonia and in mice infected with influenza A. In this study, we investigated the effect of two anticoagulants on alveolar hemorrhage after influenza A virus (IAV) infection of wild‐type mice. Wild‐type mice were anticoagulated with either warfarin or the direct thrombin inhibitor dabigatran etexilate and then infected with a mouse‐adapted influenza virus (A/Puerto Rico/8/34 H1N1). Alveolar hemorrhage was assessed by measuring hemoglobin levels in the bronchoalveolar lavage fluid (BALF). We also measured vascular permeability and viral genomes in the lung, as well as white blood cells, inflammatory mediators, and protein in BALF. Survival and body weight were monitored for 14 days after influenza A infection. In infected mice receiving either warfarin or dabigatran etexilate we observed decreased activation of coagulation in the BALF and increased alveolar hemorrhage. Warfarin but not dabigatran etexilate increased vascular permeability and mortality of influenza A‐infected mice. Anticoagulation did not affect levels of influenza A genomes, white blood cells, inflammatory mediators, or protein in the BALF. Our study indicates that systemic anticoagulation increases alveolar hemorrhage in influenza A‐infected mice.

## Introduction

Influenza, also known as the flu, causes considerable morbidity and mortality worldwide every year especially in elderly people. One of the viruses causing this infectious disease is the influenza A virus (IAV), which infects the respiratory tract, resulting in bronchitis, pneumonia, and acute respiratory distress syndrome (Bramley et al. 2012; Zhang et al. 2012). There is a high mortality associated with IAV infection especially when the disease is associated with the H1N1 strain of virus (Bramley et al. 2012).

In general, viral infections are associated with activation of coagulation and in some cases, such as Ebola, this may result in hemorrhagic fever (Geisbert et al. 2003; Antoniak et al. 2008, 2013; Huerta‐Zepeda et al. 2008; Funderburg et al. 2010; Goeijenbier et al. 2012; Antoniak and Mackman 2014). Indeed, patients with acute IAV infection exhibit a high incidence of cardiovascular events, including myocardial infarction and stroke (Smeeth et al. 2004). We and others reported that IAV infection activates the coagulation system in the airspace (Zhang et al. 2012; Antoniak et al. 2016b). Despite this local activation of coagulation, alveolar hemorrhages are observed in mice infected with IAV and also in patients with influenza pneumonia (Gilbert et al. 2010; Zhang et al. 2012; von Ranke et al. 2013; Antoniak et al. 2016b). We recently reported that expression of the procoagulant protein tissue factor (TF) in lung epithelial cells maintains lung hemostasis after IAV infection (Antoniak et al. 2016b).

Anticoagulants are widely prescribed drugs that are used to prevent or treat venous thromboembolism (VTE), and to prevent stroke in patients with atrial fibrillation and mechanical prosthetic heart valves (Thaler et al. 2015). Vitamin K antagonists (VKAs), such as warfarin, are the most commonly used oral anticoagulant drugs. They exert their anticoagulant effect by suppressing the gamma‐carboxylation of the vitamin K‐dependent procoagulant factors prothrombin, factor VII, factor IX, and factor X (Witt et al. 2016), as well as anticoagulant factors protein C and protein S. Although VKAs are highly effective, they have a narrow therapeutic range, interact with food and other drugs, require regular monitoring, and need frequent dose adjustments. More recently a new class of oral anticoagulants, the direct‐acting oral anticoagulants (DOACs), has been introduced for clinical use as an alternative to warfarin and other VKAs (Burnett et al. 2016). Dabigatran etexilate (DE) directly inhibits thrombin, whereas rivaxobaban, apixaban, and edoxaban all inhibit factor Xa. Their pharmacological profile is characterized by a rapid onset of action, a relatively short half‐life, less drug or food interactions, and they can be used in fixed doses without the need of routine monitoring.

In this study, we hypothesized that mice treated with anticoagulants would have increased alveolar hemorrhage after IAV infection. We investigated the effect of two different anticoagulants, warfarin and DE, on alveolar hemorrhage and survival in wild‐type (WT) mice after IAV infection.

## Material and Methods

### Mice

We used 8‐ to 10‐week‐old male WT (C57Bl/6J) mice for all experiments. All experimental protocols were approved by the University of North Carolina‐Chapel Hill's Institutional Animal Care and Use Committee.

### Influenza A virus

A mouse‐adapted strain of influenza A/Puerto Rico/8/1934 (H1N1, PR/8) (ATCC, Manassas, VA) was propagated in 10‐ to 12‐day‐old embryonated chicken eggs and the virus was purified as described (Antoniak et al. 2013). A hemagglutination assay was used to measure viral titers (Antoniak et al. 2013).

### Infection of mice with influenza A and outcome measures

We infected mice intranasally with a dose of 0.02 hemagglutination units (HAU) in 50 *μ*L phosphate‐buffered saline (PBS) (Antoniak et al. 2013, 2016b). After IAV infection, survival and body weights were analyzed up to 14 days. As per our animal protocol, mice with a ≥ 25% loss of initial body weight were euthanized. Lungs and bronchoalveolar lavage fluid (BALF) samples were collected 7 days after infection.

### Anticoagulation of mice

WT mice were given either 2 or 4 *μ*g/mL of warfarin (Sigma‐Aldrich, St. Louis, MO) via drinking water for 3 days and then infected with IAV. Water was refreshed every other day. For inhibition of thrombin, mice were fed a chow containing DE (10 mg/g chow) as described (Antoniak et al. 2016, 2013).

### Coagulation parameters

For measuring the prothrombin time (PT), 50 *μ*L of PT reagent containing calcium chloride (Thromboplastin‐D, Pacific Hemostasis, Middletown, VA) was added to 25 *μ*L of plasma. For measuring the activated partial thromboplastin time (aPTT), 25 *μ*L of aPTT reagent (TriniCLOT aPTT S, Tcoag, Wicklow, Ireland) was mixed with 25 *μ*L of plasma and 25 *μ*L of 0.025mol/L calcium chloride. Clotting times were measured using a STart4 coagulation analyzer (Diagnostica Stago, Parsippany, NJ). Bleeding times were measured by saphenous vein injury model as described (Monroe and Hoffman 2014), and represented as an average hemostasis time after clot disruptions. Levels of thrombin‐antithrombin complexes (TATc) in BALF supernatant were quantified by ELISA (TAT Enzygnost Micro Kit; Dade Behring, Deerfield, IL).

### Bronchoalveolar lavage

After euthanizing the mice, BALF was collected using 3 × 900 *μ*L of ice‐cold PBS. Cells were isolated from the BALF by centrifugation (500 × g, 20 min, 4°C) (Antoniak et al. 2013, 2016b). The cells were resuspended in 200 *μ*L of ice‐cold PBS. Levels of white blood cells (WBC) and hemoglobin in the resuspended cell pellet samples were determined using a Hemavet 950 (Drew Scientific, Miami Lakes, FL). Protein and albumin concentrations in BALF was quantified with the RC DC^™^ Protein Assay (Bio‐Rad Laboratories, Hercules, CA) and the Mouse Albumin ELISA Quantitation Set (Bethyl Laboratories, Montgomery, TX), respectively.

### RNA isolation and RT‐PCR

Total lung RNA was extracted using TRIzol (Life Technologies, Carlsbad, CA). RNA was reverse transcribed into cDNA using the iScript^™^ RT Supermix (Bio‐Rad) and the levels of H1N1 PR/8 genomes were quantified by real‐time PCR using SSoFast^™^ Probes Supermix and a Bio‐Rad cycler (Bio‐Rad) as described (Antoniak et al. 2016, 2013, 2016a,b). *Rpl4* was used for normalization. Real‐time PCR primer/probes were purchased from Integrated DNA Technologies (Coralville, IA).

### Measurement of cytokines/chemokines

The levels of mouse IL‐1*β*, Ccl2 (MCP‐1), Ccl5 (RANTES), and Cxcl10 (IP‐10) in the BALF supernatants were measured using commercially available ELISA kits (Duo‐Set, R&D Systems, Minneapolis, MN).

### Vascular Permeability

Vascular permeability in the lung after IAV infection was analyzed using the Evans Blue method (Sparkenbaugh et al. 2015). Mice were retroorbital injected with 100 *μ*L of 1% Evans Blue solution (Sigma‐Aldrich). One hour later, mice were anesthetized and the lung was perfused with PBS through the right heart ventricle. The whole lung was collected and dried for 2 days at 55°C. Dry lung tissue was minced and incubated at 37°C for 2 days with formamide (1 mL/ 100 mg lung weight, Sigma‐Aldrich) to extract the Evans Blue. The concentration of Evans Blue was calculated from absorbance readings at 620 nm of the cleared formamide supernatant. Differences in Evans Blue injection efficiency was normalized to Evans Blue concentrations in the plasma.

### Statistics

GraphPad Prism (version 5.0; GraphPad Software Inc., La Jolla, CA) was used for all statistical analyses. Data are represented as mean ± standard error of the mean. For two‐group comparison of continuous data, two‐tailed Student's *t‐*test was used. For multiple‐group comparison, data were analyzed by one‐ or two‐way ANOVA and were Bonferroni corrected for repeated measures over time. Survival rates were analyzed by Kaplan–Meier analysis and the log‐rank test was applied to compare the survival distribution between groups. A *P* value less than 0.05 was considered statistically significant.

## Results

### Anticoagulation of mice with warfarin or DE

**Figure 1:**
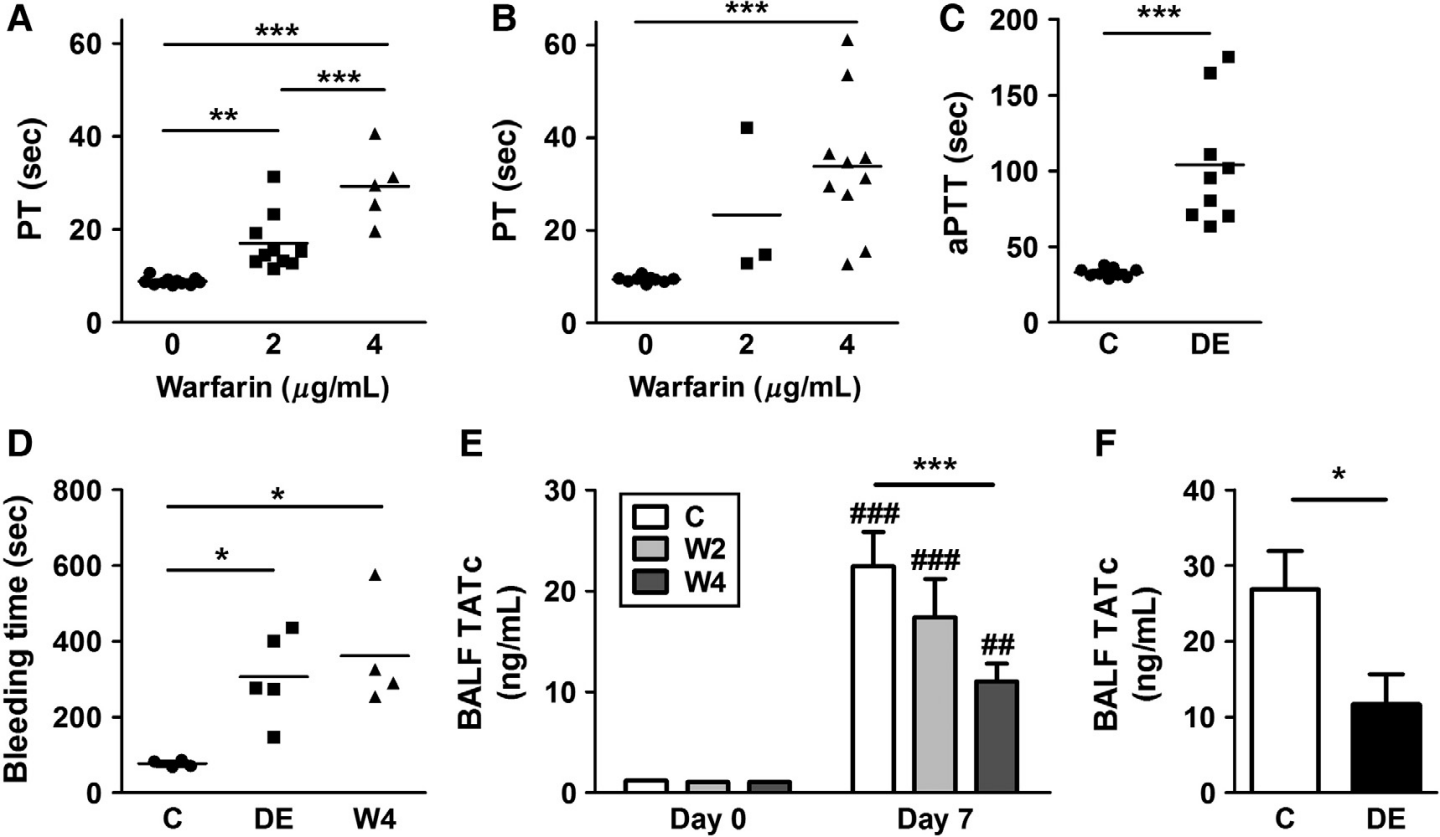
Anticoagulating mice with warfarin or dabigatran etexilate. Wild‐type (WT) mice were untreated or treated with 2 or 4 *μ*g/mL of warfarin via drinking water for 3 (A) or 7 (B) days and the level of anticoagulation was assessed by measuring the prothrombin time (PT). (C) WT mice were untreated or treated with dabigatran etexilate (DE) containing chow (10 mg/g of chow) for 3 days and the level of anticoagulation was assessed by measuring the activated partial thromboplastin time (aPTT). (D) Bleeding times of untreated WT mice or mice‐receiving warfarin (4 *μ*g/mL, drinking water) or DE (10 mg/g of chow) for 3 days were determined using the saphenous vein injury model. Data are represented as an average hemostasis time after clot disruptions. WT mice were untreated or treated with warfarin via drinking water (2 or 4 *μ*g/mL) or DE via chow (10 mg/g of chow) for 3 days before being infected with influenza A virus (IAV) intranasally. (E) Levels of thrombin–antithrombin complexes (TATc) in bronchoalveolar lavage fluid (BALF) of control (C; white), and 2 *μ*g/mL (W2; light gray) or 4 *μ*g/mL (W4; dark gray) of warfarin‐treated mice before and 7 days after IAV infection. (F) Levels of TATc in BALF of control (white) or DE‐fed (black) mice 7 days after IAV infection. Data were analyzed by one‐way ANOVA for A, B, and D, and Student's *t*‐test for C. Data are represented as mean, and statistical significances are indicated as * (*P *<* *0.05), ** (*P *<* *0.01), and *** (*P *<* *0.001) between groups. The numbers of mice used are as follows: 11, 10, and 5 for A; 10, 3, and 10 for B; 9 and 9 for C; and 4, 5, and 4 for D. Data were analyzed by two‐way ANOVA for E, and by Student's *t*‐test for F. Data are represented as mean ± SEM, and statistical significances are indicated as * (*P *<* *0.05) and *** (*P *<* *0.001) between groups, or ^##^ (*P *<* *0.01) and ^###^ (*P *<* *0.001) versus uninfected control of the respective group. The numbers of mice used are as follows: uninfected (8, 8, and 8), infected (7, 8, and 13) for E, and 5 and 5 for F.

Mice were anticoagulated with either warfarin or DE. We used two different doses of warfarin administered via the drinking water and one dose of DE administered via the chow. Warfarin significantly increased the PT in a dose‐dependent manner compared with untreated mice (Fig. 1A and B). Administration of DE significantly increased the aPTT compared with untreated mice (Fig. 1C). In addition, the high dose of warfarin and DE also significantly increased saphenous vein bleeding compared with controls (Fig. 1D). These results indicate that mice were significantly anticoagulated using both types of anticoagulants.

### Effect of warfarin and DE on activation of coagulation and alveolar hemorrhage in mice after influenza A virus infection

As expected from recent studies (Keller et al. 2006; Antoniak et al. 2016b), IAV infection of mice increased levels of TATc in the BALF indicating activation of coagulation (Fig. 1E and F). However, TATc levels were significantly lower in mice receiving either the high dose of warfarin or DE (Fig. 1E and F). The low dose of warfarin also reduced levels of TATc, but this decrease was not statistically significant (Fig. 1E). These results indicate that anticoagulation reduces the activation of coagulation in the lungs of WT mice after IAV infection.

**Figure 2:**
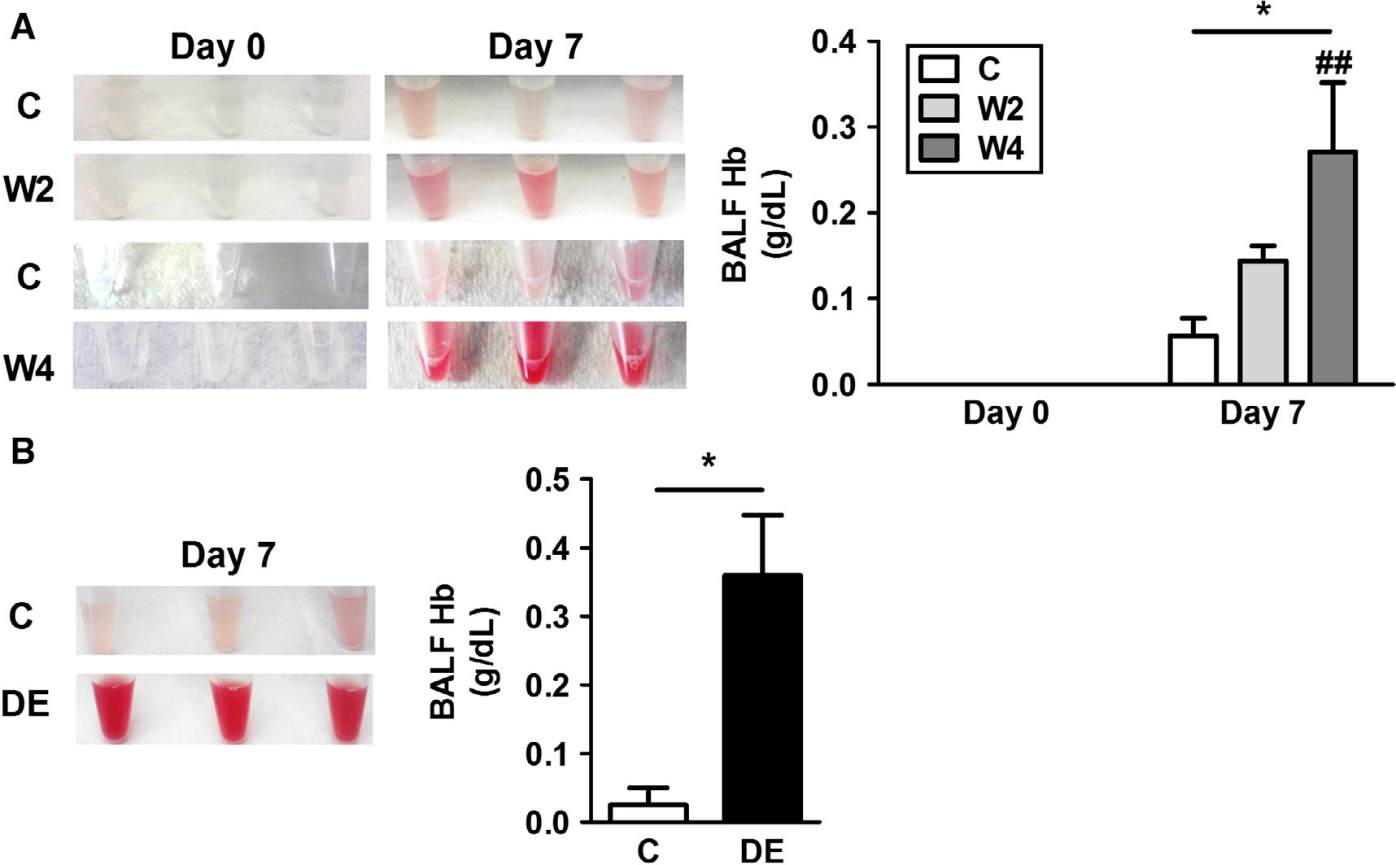
Anticoagulation with warfarin or dabigatran etexilate increases alveolar hemorrhage in mice after influenza A infection. Wild‐type mice were untreated or treated with warfarin via the drinking water (2 or 4 *μ*g/mL) or dabigatran etexilate (DE) via chow (10 mg/g of chow) for 3 days and were infected with influenza A virus (IAV) intranasally. (A) (Left) Gross appearance of bronchoalveolar lavage fluid (BALF) from three representative mice receiving with warfarin before and 7 days after IAV infection. (Right) Levels of hemoglobin (Hb) in BALF of control mice (C; white), and 2 *μ*g/mL (W2; light gray) or 4 *μ*g/mL (W4; dark gray) of warfarin‐treated mice before and 7 days after IAV infection. (B) (Left) Gross appearance of BALF from three representative mice with or without DE chow 7 days after IAV infection. (Right) Levels of Hb in BALF of control (white) or DE‐fed (black) mice at 7 days after IAV infection. Data were analyzed by two‐way ANOVA for A, and by Student's *t*‐test for B. Data are represented as mean ± SEM, and statistical significances are indicated as * (*P *<* *0.05) between groups, or ^## (^ *P *<* *0.01) versus uninfected control of the respective group. The numbers of mice used are as follows: uninfected (8, 8, and 8), infected (7, 9, and 14) for A, and 4 and 5 for B.

We and others have reported alveolar hemorrhage in mice after IAV infection (Keller et al. 2006; Schouten et al. 2010a,b; Antoniak et al. 2016b). As expected, IAV infection of WT mice led to a low level of alveolar hemorrhage (Fig. 2A and B). We observed a significant increase in alveolar hemorrhage in IAV‐infected mice receiving a high dose of warfarin or DE (Fig. 2A and B). The low dose of warfarin increased alveolar hemorrhage, but this was not statistically significant (Fig. 2B). Warfarin or DE did not increase the alveolar hemorrhage in uninfected mice (Fig. 2A). These results indicate that administration of anticoagulants to mice decreases activation of coagulation and increases alveolar hemorrhage after IAV infection.

### Effect of warfarin or DE on morbidity and mortality of mice after influenza A virus infection

**Figure 3:**
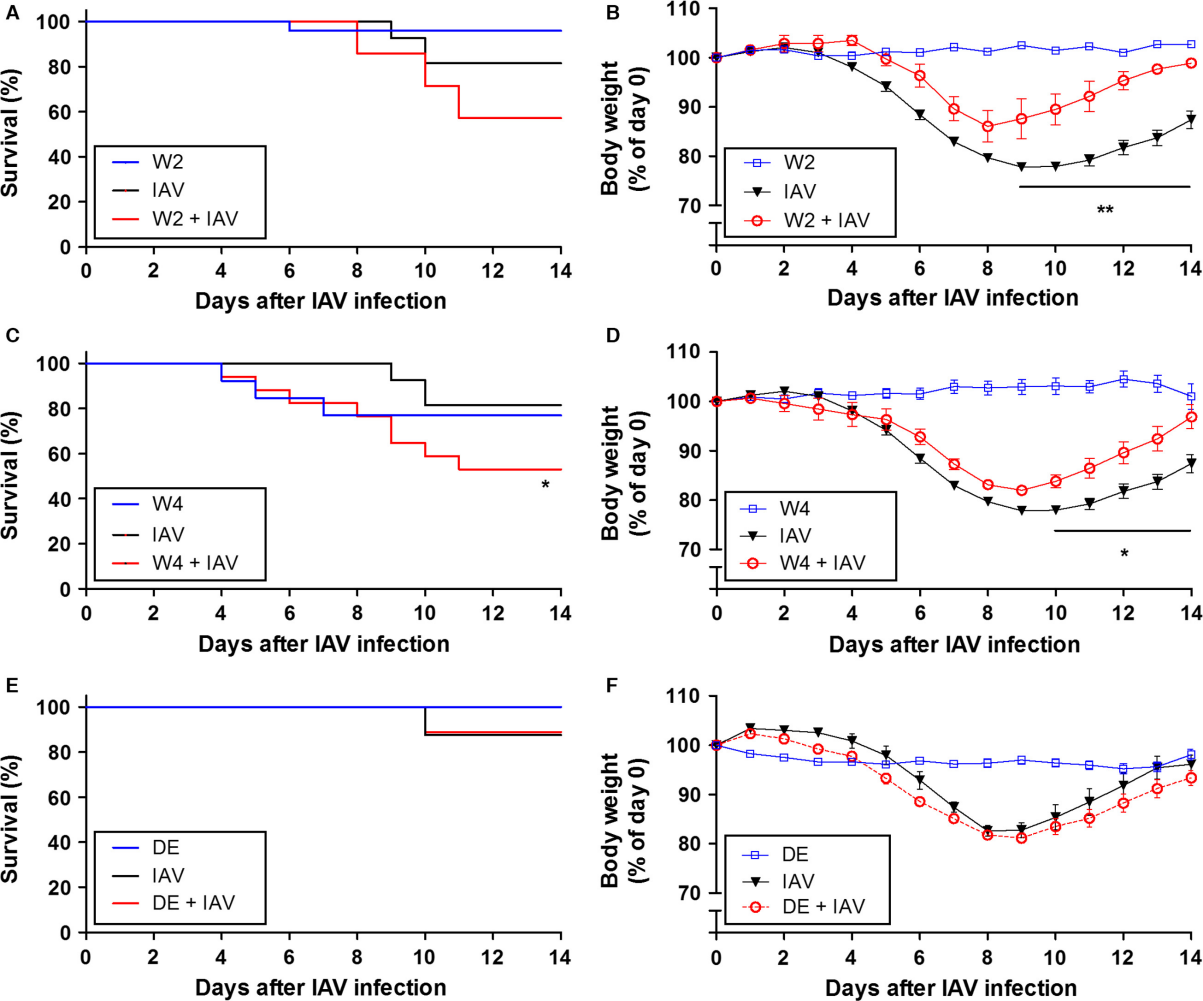
Effect of anticoagulation on morbidity and mortality in mice after influenza A infection. Wild‐type mice were untreated or treated with low‐ (2 *μ*g/mL; W2) (A and B) and high‐ (4 *μ*g/mL; W4) (C and D) dose warfarin via drinking water, or dabigatran etexilate (DE) via chow (10 mg/g of chow; DE) (E and F) for 3 days and were infected with influenza A virus (IAV) intranasally. The different groups are designated with different colors: blue indicates mice receiving anticoagulants alone; black indicates IAV alone; and red indicates mice that received anticoagulation and were infected. Body weights before infection were set to 100%. Survival (mean ± SEM; *n* = 7–27 per group) and body weight data were analyzed by log‐rank test (A, C, and E), or by two‐way ANOVA (B, D, and F). Statistical significance is indicated as * (*P *<* *0.05) between IAV‐infected mice with and without anticoagulants.

IAV infection alone led to a decrease in body weight of 10–19% of the mice at 9 days after infection and beyond (Fig. 3A, C, and E, black lines). We observed a low number of deaths in mice receiving warfarin alone (1 of 24 mice [4%] with the 2 *μ*g/mL dose of warfarin, and 3 of 13 mice [23%] with the 4 *μ*g/mL dose of warfarin) (Fig. 3A and C, blue lines). Two of the four mice that died with warfarin alone were examined post mortem; one had a subcutaneous bleed and one had both subcutaneous and gastrointestinal bleeds. Mice treated with the low dose of warfarin (2 *μ*g/mL) exhibited a trend toward increased mortality after IAV infection (3 died of 7 mice, 43%) (*P *=* *0.17), but all of these mice were euthanized due to a reduction in body weight (Fig. 3A, red line). IAV‐infected mice treated with the high dose of warfarin (4 *μ*g/mL) exhibited a significantly higher mortality (8 died of 17 mice, 48%) compared with IAV‐infected controls (*P *<* *0.05) (Fig. 3C, red line). Of the eight dead mice three were found dead between days 3 and 8. Two of the three mice that died early were examined post mortem; one had a gastrointestinal bleed and one had a lung bleed. The remaining five mice had a decrease in body weight between days 9 and 11. Interestingly, both the low and high doses of warfarin treatment accelerated the recovery of body weight compared with control mice after IAV infection (Fig. 3B and D). Administration of DE did not cause any spontaneous death and did not increase the number of mice that were euthanized due to a reduction in body weight after IAV infection (Fig. 3E). In addition. DE did not affect the weight loss and recovery of IAV‐infected mice (Fig. 3F). These results indicate that only a high dose of warfarin increases early death of mice after IAV infection, whereas both doses of warfarin affected the recovery of body weight. DE did not affect mortality or weight gain in IAV‐infected mice.

### Effect of warfarin and DE on vascular permeability in the lung after influenza A infection

**Figure 4:**
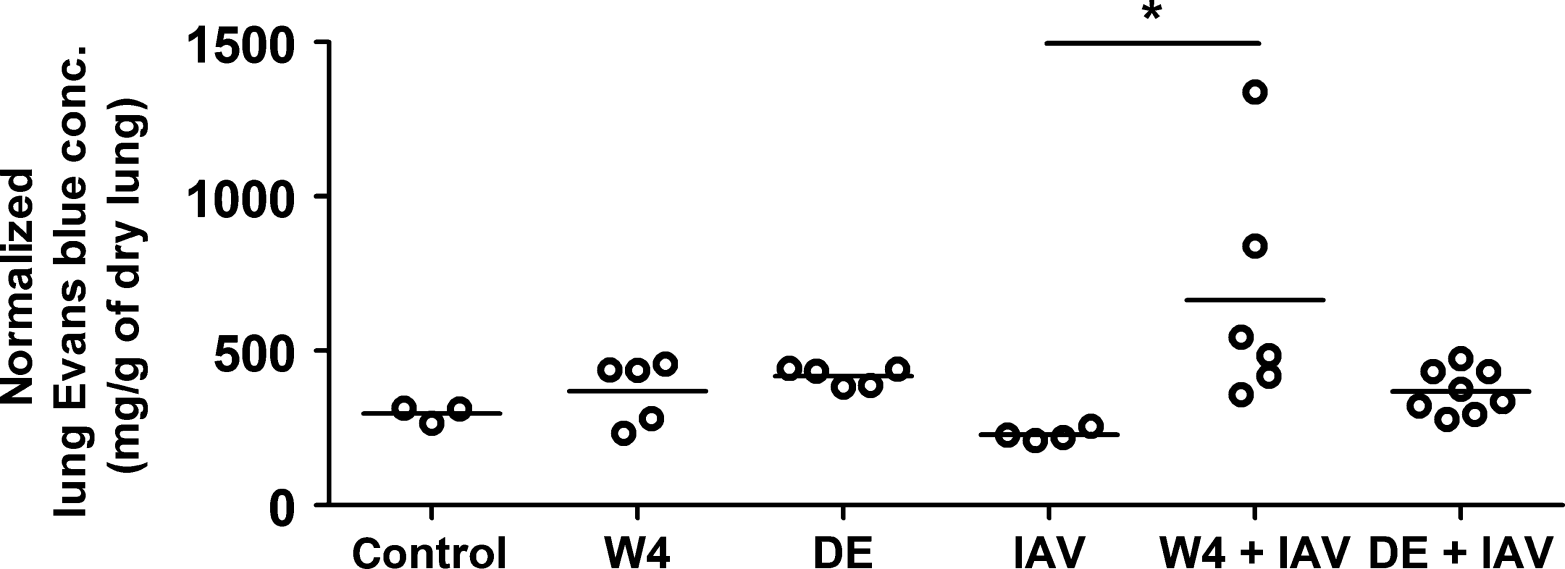
Effect of anticoagulation on vascular permeability after influenza A infection. Wild‐type mice were untreated or treated with high‐ (4 *μ*g/mL; W4) dose warfarin via drinking water, or dabigatran etexilate (DE) via chow (10 mg/g of chow) for 3 days and were infected with influenza A virus (IAV) intranasally for 3 days. Mice treated with warfarin (W4) or DE without IAV infection were used as controls. Vascular permeability was determined by measuring levels of Evans blue in the lungs. Data were analyzed by one‐way ANOVA. Statistical significance is indicated as * (*P *<* *0.05) between IAV‐infected mice with and without anticoagulants.

Warfarin but not DE reduces levels of factor VII and protein C and this may affect vascular permeability by reducing barrier protection via protease‐activated receptor 1 in the endothelium (Sen et al. 2011). Consistent with this hypothesis, we observed a significant increase in vascular permeability when IAV‐infected mice were treated with warfarin but not DE compared with IAV infection alone (Fig. 4). Importantly, anticoagulation alone had no effect on vascular permeability within the lung (Fig. 4).

### Anticoagulation does not affect viral genomes or inflammation

**Figure 5:**
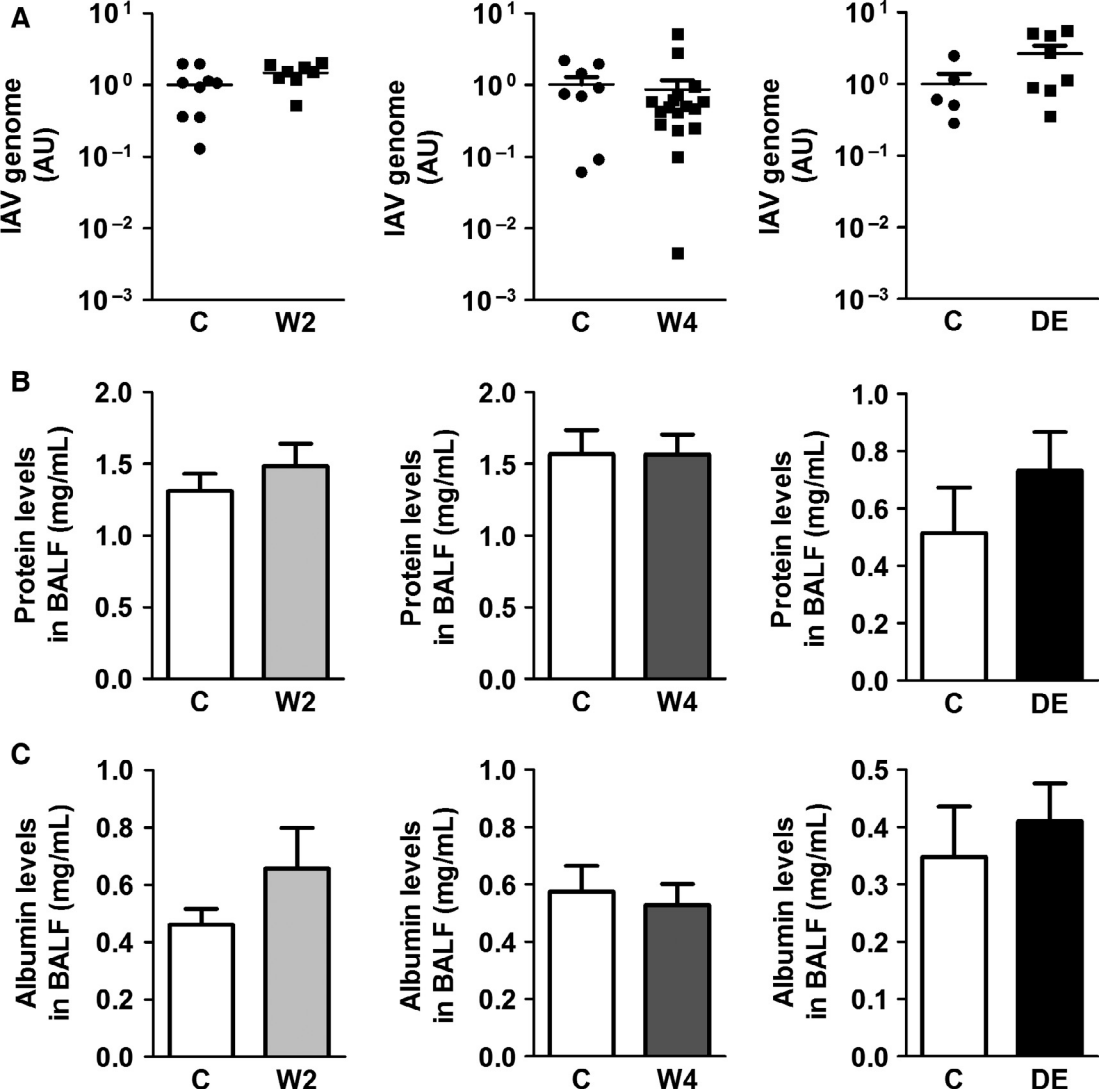
Effect of anticoagulation on IAV genomes and protein in the bronchoalveolar lavage fluid. Wild‐type mice were untreated or treated with low (2 *μ*g/mL; W2) and high (4 *μ*g/mL; W4) doses of warfarin or dabigatran etexilate (DE) for 3 days before influenza A infection. (A) Levels of influenza A genomes in the lungs were measured in control and anticoagulated mice 7 days after infection. Levels of total protein (B) and albumin (C) in the bronchoalveolar lavage fluid in control and anticoagulated mice 7 days after infection.

Recently we found that maximal levels of IAV genomes and inflammatory mediators were observed 7 days after IAV infection of mice (Antoniak et al. 2016b). Therefore, we determined if administration of anticoagulants was associated with changes in IAV genomes 7 days after infection. Anticoagulation did not change the levels of IAV genome in the lungs of the infected mice (Fig. 5A). In addition, there were no differences in the levels of total protein or albumin in the BALF of infected mice treated with the two anticoagulants (Fig. 5B and C).

**Figure 6:**
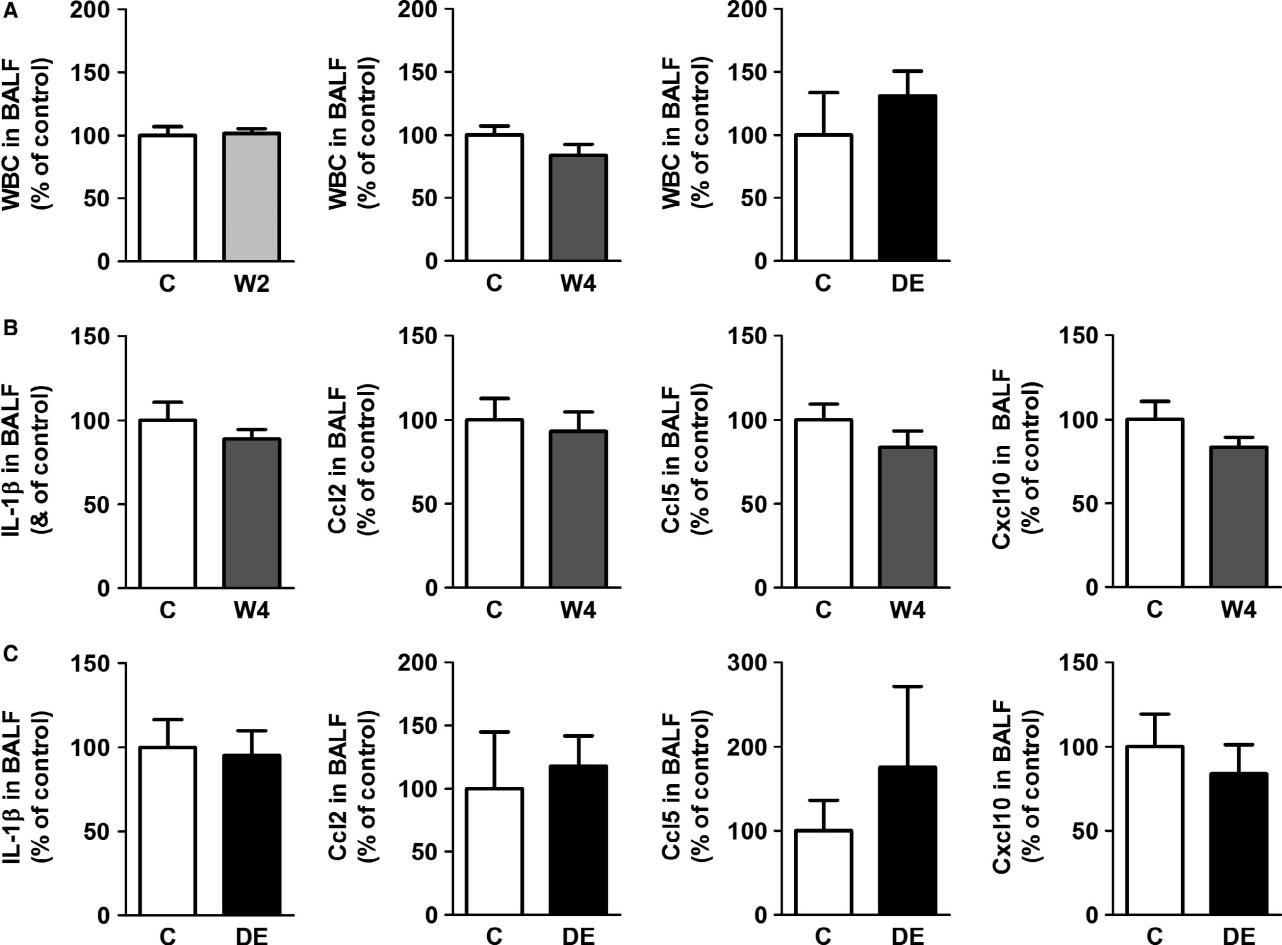
Effect of anticoagulation on white blood cell counts and inflammatory mediators in the bronchoalveolar lavage fluid. Wild‐type mice were untreated or treated with low (2 *μ*g/mL; W2) and high (4 *μ*g/mL; W4) doses of warfarin or dabigatran etexilate (DE) for 3 days before influenza A infection. Levels of white blood cells (WBC) and various cytokines/chemokines in the bronchoalveolar lavage fluid (BALF) were measured 7 days after influenza A infection. (Panel A) shows levels of WBC in the different groups. (Panels B and C) show the effect of warfarin and DE on levels of cytokines/chemokines in mice treated with high‐dose warfarin or DE, respectively. Data (mean ± SEM; *n* = 4–17 per group) were analyzed by Student's *t*‐test.

Previous studies have reported crosstalk between coagulation and inflammation (Antoniak et al.^2016^, ^2013^, 2016a; Andrade et al. 2013; Wilson et al. 2014; Azeredo et al. 2015; Yang and Tang 2016). Therefore, we determined if anticoagulation affected the recruitment of WBCs and inflammatory mediators in the BALF in IAV‐infected mice. The number of WBC or cytokines/chemokines levels in the BALF 7 days postinfection did not differ in mice treated with either warfarin or DE (Fig. 6A–C).

## Discussion

In this study, we found that administration of two different anticoagulants to mice infected with influenza A led to a significant increase in alveolar hemorrhage. The doses of warfarin that we used are similar to the therapeutic dose used in patients (Foerch et al. 2008). We observed a significant increase in mortality (48%) in IAV‐infected mice receiving a high dose of warfarin compared with IAV‐infected alone (19%) or warfarin alone (23%). In contrast, administration of DE did not increase mortality of IAV‐infected mice. Interpretation of the mortality data is complicated by the fact that warfarin alone caused death of some mice. Death due to bleeding was observed between 3 and 8 days after infection, whereas “death” due to weight loss was observed 9 days postinfection and beyond. Why do we observe an increase in death with warfarin but not with DE? One possibility is that the high dose of warfarin is producing a higher level of anticoagulation than the dose of DE. A recent study evaluated the effect of warfarin and DE in two mouse models of intracerebral hemorrhage (Lauer et al. 2011). The study reported that warfarin increased hemorrhage, whereas DE did not increase hemorrhage. The authors suggested that the incidence of microhemorrhages with warfarin‐ and DE‐treated mice is similar but that with warfarin these hemorrhages are more likely to progress to major bleedings (Lauer et al. 2011). Clinical studies have reported higher levels of major bleeding in patients treated with warfarin compared with DE (Hori et al. 2013; Salazar et al. 2014) and the use of DOACs in patients with atrial fibrillation is associated with less intracranial hemorrhage compared to warfarin (Ruff et al. 2014).

In our study, the high dose of warfarin and DE produced similar bleeding in a saphenous vein model, and a similar reduction in TATc in the BALF of IAV‐infected mice. In addition, levels of alveolar hemorrhage in IAV‐infected mice were increased to a similar extent with both anticoagulants. These results suggest that the levels of anticoagulation are comparable with the two anticoagulants. Warfarin and DE did not affect levels of viral genomes in the lung and levels of WBC, inflammatory mediators, or protein in the BALF. Therefore, an alternative possibility is that warfarin is affecting molecules or pathways that are not affected by DE. Importantly, warfarin treatment leads to unselective reduction in the vitamin K‐dependent procoagulant factors prothrombin, VII, IX, X, and the anticoagulant factors protein C and S, whereas DE only inhibits thrombin activity. Protein C and factor VII have been shown to mediate vascular protection via the endothelial protein C receptor and protease‐activated receptor‐1 in certain disease models, such as stroke and endotoxemia (Sen et al. 2011; Zlokovic and Griffin 2011). Indeed, we found that warfarin but not DE significantly increased vascular permeability in the lung after IAV infection compared to IAV alone. Interestingly, administration of warfarin significantly increased the body weight recovery beyond 9 days with the high dose and beyond 10 days with the low dose. Currently, we do not know how warfarin is affecting body weight.

In summary, we demonstrate a critical protective role for the coagulation cascade in the lung in a model of severe influenza pneumonia. Although alveolar hemorrhage is a well‐described feature of influenza pneumonia, studies on the impact of anticoagulant therapy on the severity of illness and outcomes from influenza are lacking. Our results suggest that chronic anticoagulation with warfarin or other anticoagulants, such as DE, could contribute to more severe alveolar hemorrhage in the setting of influenza pneumonia.

## Conflict of interest

The authors declare no competing financial interests.

## References

[phy213071-bib-0001] Andrade, B. B. , K. H. Hullsiek , D. R. Boulware , A. Rupert , M. A. French , K. Ruxrungtham , et al. , and Group IS . 2013 Biomarkers of inflammation and coagulation are associated with mortality and hepatitis flares in persons coinfected with HIV and hepatitis viruses. J. Infect. Dis. 207:1379–1388. 23335804 10.1093/infdis/jit033 PMC3610421

[phy213071-bib-0002] Antoniak, S. , and N. Mackman . 2014 Multiple roles of the coagulation protease cascade during virus infection. Blood 123:2605–2613. 24632711 10.1182/blood-2013-09-526277 PMC3999750

[phy213071-bib-0003] Antoniak, S. , K. Tatsumi , M. Bode , S. Vanja , J. C. Williams , and N. Mackman .2016 Protease‐activated receptor‐1 enhances poly I:C induction of the anti‐viral response in macrophages and mice. J. Innate. Immun. in press. doi: 10.1159/000450853. 10.1159/000450853 PMC5330934 27820939

[phy213071-bib-0004] Antoniak, S. , U. Boltzen , A. Riad , A. Kallwellis‐Opara , M. Rohde , A. Dorner , et al. 2008 Viral myocarditis and coagulopathy: increased tissue factor expression and plasma thrombogenicity. J. Mol. Cell. Cardiol. 45:118–126. 18495150 10.1016/j.yjmcc.2008.03.013

[phy213071-bib-0005] Antoniak, S. , A. P. 3rd Owens , M. Baunacke , J. C. Williams , R. D. Lee , A. Weithauser , et al. 2013 PAR‐1 contributes to the innate immune response during viral infection. J. Clin. Invest. 123:1310–1322. 23391721 10.1172/JCI66125 PMC3582138

[phy213071-bib-0006] Antoniak, S. , J. C. Cardenas , L. J. Buczek , F. C. Church , N. Mackman , and R. Pawlinski . 2016a Protease‐activated receptor 1 contributes to angiotensin II‐induced cardiovascular remodeling and inflammation. Cardiology 136:258–268. 27880950 10.1159/000452269 PMC5334284

[phy213071-bib-0007] Antoniak, S. , K. Tatsumi , Y. Hisada , J. J. Milner , S. D. Neidich , C. M. Shaver , et al. 2016b Tissue factor deficiency increases alveolar hemorrhage and death in influenza A virus‐infected mice. J. Thromb. Haemost. 14:1238–1248. 26947929 10.1111/jth.13307 PMC5892427

[phy213071-bib-0008] deAzeredo, E. L. , R. Q. Monteiro , and de‐Oliveira Pinto L. M. . 2015 Thrombocytopenia in dengue: interrelationship between virus and the imbalance between coagulation and fibrinolysis and inflammatory mediators. Mediators Inflamm. 2015:313842. 25999666 10.1155/2015/313842 PMC4427128

[phy213071-bib-0009] Bramley, A. M. , S. Dasgupta , J. Skarbinski , L. Kamimoto , A. M. Fry , L. Finelli , et al. , and Pandemic Influenza AVHIT . 2012 Intensive care unit patients with 2009 pandemic influenza A (H1N1pdm09) virus infection ‐ United States, 2009. Influenza Other Respir. Viruses 6:e134–e142. 22672249 10.1111/j.1750-2659.2012.00385.x PMC4941711

[phy213071-bib-0010] Burnett, A. E. , C. E. Mahan , S. R. Vazquez , L. B. Oertel , D. A. Garcia , and J. Ansell . 2016 Guidance for the practical management of the direct oral anticoagulants (DOACs) in VTE treatment. J. Thromb. Thrombolysis 41:206–232. 26780747 10.1007/s11239-015-1310-7 PMC4715848

[phy213071-bib-0011] Foerch, C. , K. Arai , G. Jin , K. P. Park , S. Pallast , K. van Leyen , et al. 2008 Experimental model of warfarin‐associated intracerebral hemorrhage. Stroke 39:3397–3404. 18772448 10.1161/STROKEAHA.108.517482 PMC3712841

[phy213071-bib-0012] Funderburg, N. T. , E. Mayne , S. F. Sieg , R. Asaad , W. Jiang , M. Kalinowska , et al. 2010 Increased tissue factor expression on circulating monocytes in chronic HIV infection: relationship to in vivo coagulation and immune activation. Blood 115:161–167. 19828697 10.1182/blood-2009-03-210179 PMC2808148

[phy213071-bib-0013] Geisbert, T. W. , H. A. Young , P. B. Jahrling , K. J. Davis , E. Kagan , and L. E. Hensley . 2003 Mechanisms underlying coagulation abnormalities in ebola hemorrhagic fever: overexpression of tissue factor in primate monocytes/macrophages is a key event. J. Infect. Dis. 188:1618–1629. 14639531 10.1086/379724

[phy213071-bib-0014] Gilbert, C. R. , K. Vipul , and M. Baram . 2010 Novel H1N1 influenza A viral infection complicated by alveolar hemorrhage. Respir. Care 55:623–625. 20420734

[phy213071-bib-0015] Goeijenbier, M. , M. van Wissen , C. van de Weg , E. Jong , V. E. Gerdes , J. C. Meijers , et al. 2012 Review: viral infections and mechanisms of thrombosis and bleeding. J. Med. Virol. 84:1680–1696. 22930518 10.1002/jmv.23354 PMC7166625

[phy213071-bib-0016] Hori, M. , S. J. Connolly , J. Zhu , L. S. Liu , C. P. Lau , P. Pais , et al. 2013 Dabigatran versus warfarin: effects on ischemic and hemorrhagic strokes and bleeding in Asians and non‐Asians with atrial fibrillation. Stroke 44:1891–1896. 23743976 10.1161/STROKEAHA.113.000990

[phy213071-bib-0017] Huerta‐Zepeda, A. , C. Cabello‐Gutierrez , J. Cime‐Castillo , V. Monroy‐Martinez , M. E. Manjarrez‐Zavala , M. Gutierrez‐Rodriguez , et al. 2008 Crosstalk between coagulation and inflammation during Dengue virus infection. Thromb. Haemost. 99:936–943. 18449425 10.1160/TH07-08-0438

[phy213071-bib-0018] Keller, T. T. , K. F. van der Sluijs , M. D. de Kruif , V. E. Gerdes , J. C. Meijers , S. Florquin , et al. 2006 Effects on coagulation and fibrinolysis induced by influenza in mice with a reduced capacity to generate activated protein C and a deficiency in plasminogen activator inhibitor type 1. Circ. Res. 99:1261–1269. 17068293 10.1161/01.RES.0000250834.29108.1a

[phy213071-bib-0019] Lauer, A. , F. A. Cianchetti , E. M. Van Cott , F. Schlunk , E. Schulz , W. Pfeilschifter , et al. 2011 Anticoagulation with the oral direct thrombin inhibitor dabigatran does not enlarge hematoma volume in experimental intracerebral hemorrhage. Circulation 124:1654–1662. 21911784 10.1161/CIRCULATIONAHA.111.035972 PMC3724228

[phy213071-bib-0020] Monroe, D. M. , and M. Hoffman . 2014 A mouse bleeding model to study oral anticoagulants. Thromb. Res. 133(Suppl 1):S6–S8. 24759147 10.1016/j.thromres.2014.03.003 PMC4014069

[phy213071-bib-0021] von Ranke, F. M. , G. Zanetti , B. Hochhegger , and E. Marchiori . 2013 Infectious diseases causing diffuse alveolar hemorrhage in immunocompetent patients: a state‐of‐the‐art review. Lung 191:9–18. 23128913 10.1007/s00408-012-9431-7 PMC7102311

[phy213071-bib-0022] Ruff, C. T. , R. P. Giugliano , E. Braunwald , E. B. Hoffman , N. Deenadayalu , M. D. Ezekowitz , et al. 2014 Comparison of the efficacy and safety of new oral anticoagulants with warfarin in patients with atrial fibrillation: a meta‐analysis of randomised trials. Lancet 383:955–962. 24315724 10.1016/S0140-6736(13)62343-0

[phy213071-bib-0023] Salazar, C. A. , D. del Aguila , and E. G. Cordova . 2014 Direct thrombin inhibitors versus vitamin K antagonists for preventing cerebral or systemic embolism in people with non‐valvular atrial fibrillation. Cochrane Database Syst. Rev.:CD009893. doi: 10.1002/14651858.CD009893.pub2. 24677203 10.1002/14651858.CD009893.pub2 PMC8928929

[phy213071-bib-0024] Schouten, M. , K. F. Sluijs , B. Gerlitz , B. W. Grinnell , J. J. Roelofs , M. M. Levi , et al. 2010a Activated protein C ameliorates coagulopathy but does not influence outcome in lethal H1N1 influenza: a controlled laboratory study. Crit. Care 14:R65. 20398279 10.1186/cc8964 PMC2887187

[phy213071-bib-0025] Schouten, M. , dervan Sluijs K. F. , J. J. Roelofs , M. Levi , C. Van't Veer , and dervan Poll T. . 2010b Factor V Leiden mutation does not affect coagulopathy or outcome in lethal H1N1 influenza. Eur. Respir. J. 36:1346–1354. 20413539 10.1183/09031936.00204909

[phy213071-bib-0026] Sen, P. , R. Gopalakrishnan , H. Kothari , S. Keshava , C. A. Clark , C. T. Esmon , et al. 2011 Factor VIIa bound to endothelial cell protein C receptor activates protease activated receptor‐1 and mediates cell signaling and barrier protection. Blood 117:3199–3208. 21252088 10.1182/blood-2010-09-310706 PMC3062318

[phy213071-bib-0027] Smeeth, L. , S. L. Thomas , A. J. Hall , R. Hubbard , P. Farrington , and P. Vallance . 2004 Risk of myocardial infarction and stroke after acute infection or vaccination. N. Engl. J. Med. 351:2611–2618. 15602021 10.1056/NEJMoa041747

[phy213071-bib-0028] Sparkenbaugh, E. M. , P. Chantrathammachart , S. Wang , W. Jonas , D. Kirchhofer , D. Gailani , et al. 2015 Excess of heme induces tissue factor‐dependent activation of coagulation in mice. Haematologica 100:308–314. 25596265 10.3324/haematol.2014.114728 PMC4349268

[phy213071-bib-0029] Thaler, J. , I. Pabinger , and C. Ay . 2015 Anticoagulant treatment of deep vein thrombosis and pulmonary embolism: the present state of the art. Front. Cardiovasc. Med. 2:30. 26664901 10.3389/fcvm.2015.00030 PMC4671349

[phy213071-bib-0030] Wilson, E. M. , A. Singh , K. H. Hullsiek , D. Gibson , W. K. Henry , K. Lichtenstein , et al. , and Study to Understand the Natural History of HIVAitEoETI . 2014 Monocyte‐activation phenotypes are associated with biomarkers of inflammation and coagulation in chronic HIV infection. J. Infect. Dis. 210: 1396–1406. 24813472 10.1093/infdis/jiu275 PMC4207864

[phy213071-bib-0031] Witt, D. M. , N. P. Clark , S. Kaatz , T. Schnurr , and J. E. Ansell . 2016 Guidance for the practical management of warfarin therapy in the treatment of venous thromboembolism. J. Thromb. Thrombolysis 41:187–205. 26780746 10.1007/s11239-015-1319-y PMC4715850

[phy213071-bib-0032] Yang, Y. , and H. Tang . 2016 Aberrant coagulation causes a hyper‐inflammatory response in severe influenza pneumonia. Cell. Mol. Immunol. 13:432–442. 27041635 10.1038/cmi.2016.1 PMC4947825

[phy213071-bib-0033] Zhang, Y. , H. Sun , L. Fan , Y. Ma , Y. Sun , J. Pu , et al. 2012 Acute respiratory distress syndrome induced by a swine 2009 H1N1 variant in mice. PLoS ONE 7:e29347. 22235288 10.1371/journal.pone.0029347 PMC3250439

[phy213071-bib-0034] Zlokovic, B. V. , and J. H. Griffin . 2011 Cytoprotective protein C pathways and implications for stroke and neurological disorders. Trends Neurosci. 34:198–209. 21353711 10.1016/j.tins.2011.01.005 PMC3491752

